# Feasibility of Upper Gastrointestinal Examination in Home Care Setting with a Magnetically Assisted Capsule Endoscopy System: A Retrospective Study

**DOI:** 10.3390/healthcare9050577

**Published:** 2021-05-13

**Authors:** Yang-Chao Lin, Ching-Lin Chen, Yi-Wei Kao, Chi-Yang Chang, Ming-Chih Chen, Chih-Kuang Liu

**Affiliations:** 1 Department of Gastroenterology and Hepatology, Fu Jen Catholic University Hospital, New Taipei 242, Taiwan; yangchao.lin@gmail.com (Y.-C.L.); chiyang1112@gmail.com (C.-Y.C.); 2 Graduate Institute of Business Administration, Fu Jen Catholic University, New Taipei 242, Taiwan; a1554@tpech.gov.tw (C.-L.C.); kyw498762030@gmail.com (Y.-W.K.); 081438@mail.fju.edu.tw (M.-C.C.); 3 Taipei City Hospital, Zhong-Xing Branch, Taipei 10341, Taiwan; 4 Department of Urology, Fu Jen Catholic University Hospital, New Taipei 242, Taiwan

**Keywords:** magnetic assisted, capsule endoscopy, upper gastrointestinal, home care, peptic ulcer, acid reflux

## Abstract

The magnetic assisted capsule endoscope (MACE) with a hand-held magnetic field navigator (MFN) for upper gastrointestinal examination achieved satisfactory results in a healthy volunteer study. We evaluated the feasibility of upper gastrointestinal examination in the home care setting with the MACE system. Home care patients with upper gastrointestinal symptoms that received an MACE exam were enrolled in the study. MACE procedure time; completeness of observation of important anatomical landmarks; endoscopic diagnosis; patient tolerance during the procedure; and patient data, including age, sex, comorbidities, symptoms, body weight, and height, were retrieved from hospital information system for data analysis. A total of 16 participants were enrolled with a mean age 74.3 ± 15.4 years (47 to 99 years). One patient failed to swallow the capsule and was excluded. The average procedure time was 23.7 ± 10.0 min (14.1 to 42.5 min) to complete each endoscopic exam for the remaining 15 patients. The overall maneuverability in the esophagus, stomach, and duodenum was 93.75%, 87.5%, and 75%, respectively. Overall completeness in the aforementioned regions was 93.75%, 81.25%, and 75%, respectively. No severe adverse events were noted. The results clearly demonstrate the promise of using this MACE system to perform endoscopic examination outside the hospital for patients confined to the community and home.

## 1. Introduction

Two decades have passed since the first commercial capsule endoscopy (CE) was launched in 2000 [1,2]. CE provides an alternative method for inspecting gastrointestinal tracts without discomfort or need for sedation. However, due to lack of operator-controlled navigation, wireless CE is ineffective for observing saccular organs, such as the stomach or duodenum.

In 2019, Insight Medical Solutions (IMS) introduced the IMS MACE system, a novel magnetic-assisted capsule endoscope (MACE) system with a hand-held magnetic field navigator (MFN). This technology was cleared by the Taiwan Food and Drug Administration for use in Taiwan. Unlike wireless capsule endoscopy in the past [3,4], InsightEyes has two distinguishing features: each capsule endoscope is wired by a thin (1 mm) and soft cable, which is used for imaging data and power transmission. In addition, when applied with an external magnetic field, the cable plays an important role in maintaining different postures of the capsule and enables complete upper GI examination. Moreover, the relatively compact size of the system makes it highly portable and thus possible for physicians to perform upper GI examination in various care settings and even outside the hospital if necessary.

Unlike most other in-building magnetic-controlled capsule endoscopy systems developed in recent decades [5,6,7,8], the IMS MACE system is highly portable and thus provides a solution for physicians to perform upper GI endoscopy for patients outside hospital or clinic settings. It is known that peptic ulcer disease and esophageal reflux are common in the senior population [9]. Seniors are prone to co-morbidities such as cardiovascular disease, cerebral vascular disease, degenerative arthritis, and osteoporosis and, as a result, have higher likelihood of taking anti-platelet agents, non-steroidal anti-inflammatory drugs, and bisphosphonates. This may result in acid-related disorders with symptoms such as acid reflux, dyspepsia, epigastric pain or anorexia, and the underlying pathology is mostly neglected unless alarm symptoms develop. Unfortunately, elderly patients with peptic ulcer disorders might have slight or atypical presentation, thus resulting in delayed diagnosis [10]. The current standard of care prevents physicians from performing gastrointestinal endoscopy outside the hospital due to the issue of portability of the endoscopy system. Therefore, senior or disabled patients who have difficulty visiting hospitals or clinics when they encounter gastrointestinal or acid-related symptoms are often underdiagnosed.

In 2015, the Taiwan National Health Institute launched the “Home-based Medical Integration Program”. Patients with physical or mental disabilities who are older than 65 with certain physical disabilities and confined to their homes are eligible to apply for the medical integration program. Medical teams of the corresponding hospital provide medical services, such as blood checks, physical examinations, electrocardiograms, abdominal ultrasounds, and medicine prescriptions for patients at their residency. A previous study of this MACE system showed satisfactory results of maneuverability, visual completeness, safety, and tolerance in the examination of upper gastrointestinal tracts of healthy volunteers [11]. Motivated by this program and the identified gap of elderly and disabled patients being underdiagnosed for GI disorders, we launched a study to evaluate the feasibility, tolerability, and safety of a portable MACE system for examining gastrointestinal tracts of senior and home care patients at their residence.

## 2. Materials and Methods

### 2.1. Participants

Among the 256 registered patients in our “Home-based Medical Integration Program”, patients who had complaints of dyspepsia, epigastralgia, acid reflux, heartburn, or anorexia since 2015 were reported to our gastroenterologist by nursing staff. The physician first reviewed the medical records and discussed the physical conditions of the patient with community nurses in charge. Then, these patients were arranged for MACE examination if eligible. Patients with known difficulty swallowing, gastric surgery history, any malignancy history, gastrointestinal obstruction, pacemaker implants, or metal implants were excluded from the study. A total of 16 home care patients were enrolled to receive MACE examinations between March 2020 and May 2020.

### 2.2. IMS MACE System (Insight Medical Solutions Crop, Hsinchu City, Taiwan)

**Figure 1:**
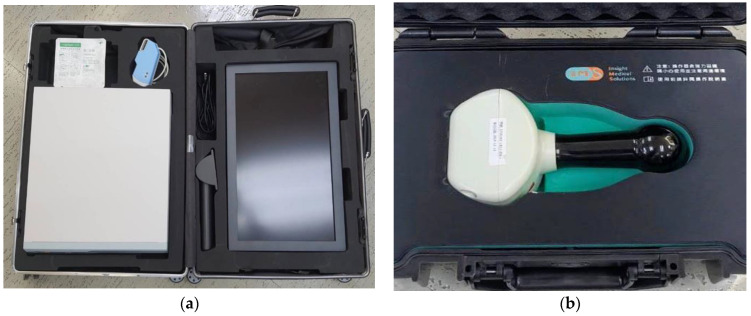
(**a**) Image processor, LCD monitor and InsightEyes EGD, packed in a roller suitcase. (**b**) Hand-held magnetic field navigator.

The MACE system was packed in an aluminum roller suitcase, which contained disposable InsightEyes^®^ EGD, a hand-held magnetic field navigator (MFN), an Aries E500i image processing unit, and a 21-inch LCD monitor (Figure 1).

### 2.3. Patient Preparation

All patients were required to fast for at least 6 hours prior to the exam. A total of 400 mg of N-acetylcysteine tablets for the mucolytic effect dissolved in 500 mL clear water was administered one hour before examination [12,13,14]. Additional clear water intake during MACE examination was encouraged if some mucus or bubbles interfered with the observed mucosa.

Upon arriving at the patient’s residency, our staff assembled the MACE system, while nursing staff explained the procedure and provided instructions regarding how to swallow the capsule.

### 2.4. MACE Examination

Written consent was obtained before examination, and MACE examinations were carried out in the patients’ homes. The medical team included an endoscopist experienced with MACE examinations, a community care nurse who was in-charge of the patient, and an endoscopy technician who aided the patient during the procedure if necessary. Patients were instructed to pinch the string of the CE and put the capsule at the base of the tongue. Patients would then swallow a mouthful of water with the capsule to force the capsule down the esophagus. If the patient has difficulty swallowing the capsule, our nurse helped the patient to place the capsule at the base of the tongue and slightly push the capsule deeper down the throat. During examination, the patient could speak to the doctor if necessary, and the physician could ask the patient to change posture or drink more water. The endoscopist controlled the orientation and movements of the capsule using a hand-held MFN to observe all necessary parts of the upper gastrointestinal tracts. When the examination ended, the patient or our nurse was instructed to slowly pull out the capsule. The esophagus was observed for a second time during withdrawal of the capsule.

The environmental requirement for the MACE procedure is as follows: a chair or bed was used to complete the MACE procedure; patients were instructed to sit on a chair or bed to swallow the CE, and then lie down on a bed or couch after the CE entered the stomach; patients had to change to the left or right decubitus position as necessary when the doctor manipulated the CE.

No intramuscular injection of hyoscine-N-butylbromide or spray of xylocaine on the throat was needed. No vacuum machine or CO_2_ pump, requirements for traditional esophagogastroduodenoscopy (EGD), was required for the MACE exam.

## 3. Data Collection

MACE procedure time, completeness of observation of important anatomical landmarks, endoscopic diagnosis, and patient tolerance during endoscopy were our primary data of interest for generating descriptive statistics. The MACE exam report and patients data, including age, sex, comorbidities, symptoms, body weight, and height, were retrieved from the hospital information system with permission from the Taipei City Hospital Research Ethics Committee (TCHIRB-10906010-E).

### Statistical Analysis

Descriptive analysis was applied to address the demographic features of our patients, quality of the MACE examination, diagnostic outcomes, safety, and the tolerability of patients. Maneuverability is defined as the ability of the capsule to be positioned at the specific landmark during examination (%maneuverability = number of successful positioning/total study number × 100%). Completeness was defined as the rate of detailed observation of targeted landmarks (%completeness = number successful observation/total study number × 100%). A satisfactory result is defined as more than 90% patients completing the MACE examination with completeness of greater than 90%. In addition, no major complications or equipment malfunction during examination were observed during the examinations.

## 4. Results

Sixteen home care patients (eight female and eight male) were enrolled in this novel MACE study. The mean age was 74.3 ± 15.4 years (47 to 99 years). One female patient (case 16, age 83) failed to swallow the capsule by herself and with our assistance, so she was excluded from this study. It took an average of 23.7 ± 10.0 min (14.1 to 42.5 min) to complete each endoscopic study for the remaining 15 patients. An average of 2.2 attempts (1 to 5 attempts) per patient were taken to successfully swallow the capsule endoscope with or without assistance. Demographics are shown in Table 1.

### 4.1. Maneuverability and Completeness of Examination

One of the sixteen patients failed to swallow the capsule. On the intention-to-treat basis, the maneuverability and completeness in the esophagus were both 93.75% (see Table 2). There was no difficulty in positioning and observing the gastric body, antrum, pylorus, and angularis using the string-pulled capsule (93.75% maneuverability and 93.75% completeness). However, we failed to observe cardia and fundus in 3 of the 16 patients (18.75%). One case was due to failed positioning of capsule, and the other case was due to mucus interference.

The capsule endoscope failed to pass through the pylorus in four patients (25%). One patient had marked pyloric stenosis, and the capsule had difficulty traversing to the duodenum in two other patients. As a result, the overall maneuverability and completeness of observing the duodenum were both 75% and 75%. All capsules reached the second portion in patients whose duodenal bulbs were observed.

### 4.2. Tolerability and Safety

All patients consumed at least 500 mL of 400 mg *N*-acetylcysteine dissolved in clear water in preparation for MACE examination. Additional clear water of about 500 mL was given to each patient to facilitate maneuverability or clearance of bubbles and mucus as necessary. Of the fifteen patients that finished the MACE, none vomited during or after the procedure. Five patients complained of mild nausea and foreign body sensation in the throat. None complained of epigastric pain during examination when the physician manipulated the MFN over their abdomen. No severe adverse events were noted during examination, and all capsules were retrieved with no noticeable problems.

### 4.3. Diagnostic Results

Of the 15 patients who underwent MACE examination, 66.7% had reflux esophagitis, 46.7% had gastric erosions, 20% had gastric ulcers, and 13.3% had duodenal ulcers, while lesions like hiatus hernia and pyloric stenosis comprised of less than 10% of the patients.

## 5. Discussion

This novel medical service providing upper gastrointestinal endoscopy for home-care patients using the MACE system revealed a satisfactory preliminary result. Patients tolerated the procedure well, and no noticeable procedure-related complications during or after the endoscopic examinations were observed.

The portability of the MACE system enabled physicians to perform upper GI endoscopic examinations at the patients’ residencies, which is unprecedented. In our study, most of the patients (15 of 16 patients, 93.75%) successfully finished the MACE examination, and diagnostic results helped the physicians to pinpoint the exact diagnosis and provide recommended therapy. However, we did notice some limitations and technical issues to be solved in the near future.

**Figure 2:**
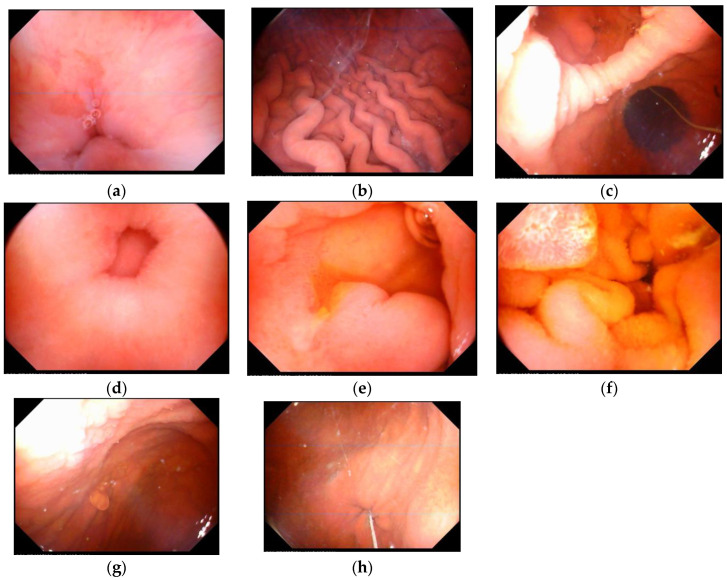
Landmark images of the upper gastrointestinal tracts by magnetic-assisted capsule endoscopy. (**a**) Esophago-gastric junction, (**b**) gastric body, (**c**) antrum and angularis, (**d**) pylorus, (**e**) duodenal bulb, (**f**) duodenal second portion, (**g**) fundus, (**h**) cardia.

Completeness of the MACE examination is one of the major considerations on whether MACE is a suitable device for examining upper GI tracts. The image quality of this MACE system was good enough for the physician to observe desired landmarks (Figure 2). The ability of detailed observation of mucosa through the endoscope was essential for the endoscopist to make a precise diagnosis. A previous study of MACE on healthy volunteers in 2017 showed 100% completeness in the esophagus, 85.2% in the stomach, and 86.2% in the duodenum [11]. Similar results were reported in a systemic review [15], and the overall completeness of observing essential landmarks of the esophagus, stomach, and duodenum were 93.75%, 81.25%, and 75%, respectively, in our study. However, cardia and fundus were not clearly observed in 18.75% of patients. Two factors might contribute to the insufficient completeness of observation: one factor is related to mucosa obscured by redundant bubbles or mucus, which may result from inadequate gastric preparation. The other is related to difficulty in maneuvering the capsule in certain patients. After reviewing the video recordings of each examination, maneuverability of the capsule to observe the cardia and fundus was achieved during examination, but it was difficult for the physicians to remove the bubbles from the surfaces of mucosa despite changes in positions or additional water intake. The postural limitation of senior patients further made it difficult to manipulate the MFN. Thus, pre-MACE gastric preparation is likely a key factor to improve the quality of capsule endoscopy. In addition, the capsule failed to pass through the pyloric ring in 4 of the 16 patients (25%), with one of the patients being found to have pyloric stenosis. It was crucial for the physician to manipulate the capsule as close as possible to the pyloric ring, waiting for gastric peristalsis while keeping magnetic traction of the capsule toward the duodenal bulb. However, MACE lacks a shaft push force like the traditional endoscope, and the real-world completeness of duodenal observation (75%) was inferior to that of a previous health pioneer study (86.1%) [11].

Adverse events of capsule endoscopy, such as rate of retention, aspiration, and procedure-related adverse events, were known to be less than 1% in a systemic review [15]. In our patients, none of the participants reported vomiting or choking during or after the MACE examination, with only 5 patients complaining of nausea and foreign body sensation in the throat. None of the reported discomforts interrupted the procedure, and every capsule was retrieved successfully after examination. The results of safety and tolerability were encouraging, because the elderly and disabled patients were especially vulnerable to choking and aspiration.

The mean procedure time was 23.7 min ± 10.0 min. The longest time to complete the examination was 42 min for a 70-year-old male, who had pyloric stenosis, and the capsule eventually failed to pass to the duodenum after several attempts. The time for the capsule to pass to the duodenum seemed to be an important determining factor on procedure time. Thus, we further divided the patients into two groups for timing analysis: the duodenal group (n = 12) and the non-duodenal group (n = 3; failed to pass the pylorus). The results were 20.75 min for the duodenal group vs. 35.33 min for the non-duodenal group, with a statistically significant difference (95% CI = 2.91–26.23, *p* = 0.02497). The sample size was small, and a future study should be conducted to further clarify if the time for the capsule to traverse the pylorus is a critical factor in determining the procedure time.

This study had several limitations: first, this is a retrospective study, which lacked a control group for comparison of all measurable parameters; second, a small study population may result in exaggerated selection bias. Thus, a prospective study with a larger sample size should be considered.

## 6. Conclusions

This is the first real-world study of the MACE system to be utilized in home care patients. The results showed that the MACE system for in-home use was satisfactory in relation to patient safety and tolerance. Observation of the esophagus was satisfactory, but maneuverability and completeness in the stomach and duodenum were less than 90% in this study. However, these results clearly demonstrate the possibility of using this MACE system to perform endoscopic examination outside the hospital setting for patients confined to the community and home.

## Figures and Tables

**Table 1:** Demographic characteristics of the patients. HTN: hypertension, DM: diabetes mellitus, CAD: coronary artery disease, OA: osteoarthritis, CVA: cerebrovascular accident, HIVD: herniated intervertebral disc, GEGRD: gastroesophageal reflux disease, HCV: hepatitis C virus, GU: gastric ulcer, CP: cerebral palsy, Pul TB: pulmonary tuberculosis, ICH: intracranial hemorrhage, BMI: body mass index.

Case	Age	Sex	Comorbidites	Reporting Symptoms	Procedure Time (min)	Time to Duodenum (min)	Attempts for Swallowing	Height (cm)	Weight (kg)	BMI
1	63	M	HTN, DM	Heart burn	30	NA	2	169	60	21.0
2	70	M	HTN, DM, CAD, OA	Hiccups	34	NA	2	155	80	33.3
3	77	F	HTN, DM, CAD, CVA, HIVD	Dyspepsia, acid reflux	18	12	1	142	72	35.7
4	70	M	HTN, DM, CVA	Dyspepsia, acid reflux	42	NA	2	160	56	21.9
5	56	M	HTN, HI, CVA, L’t Hemiplegia	Dyspepsia, acid reflux	11	3	4	165	53	19.5
6	82	F	HTN, CAD, Asthma	Dyspepsia, acid reflux	24	9	2	166	61	22.1
7	91	M	HTN, GERD, DM, HCV	Flatulence, dyspepsia	19	9	1	150	67	29.8
8	90	M	HTN, DM, GU, Gout	Dyspepsia, acid reflux	14	3	3	158	46	18.4
9	47	F	CP, Pressure sore.	Flatulence, dyspepsia	23	13	1	145	68	32.3
10	69	F	HTN, DM, CAD, OA	Dyspepsia	19	5	5	166	65	23.6
11	90	F	Glaucoma, HTN, DM, CAD	Dyspepsia	41	31	4	150	50	22.2
12	87	M	HTN, CAD, GERD, Pul TB.	Heart burn	15	11	2	159	45	17.8
13	53	M	Traffic Accident, Quadriplegia	Heart burn	18	3	1	165	70	25.7
14	62	F	ICH, R’t hemiplegia	Heart burn	33	18	1	150	60	26.7
15	99	F	HTN, DM, CAD, OA	Epigastralgia	14	5	2	162	65	24.8
16	83	F	HTN, CAD, GU	Epigastralgia	NA	NA	5	157	60	24.3

**Table 2:** Completeness and maneuverability of MACE for important landmarks of upper gastrointestinal tracts. Completeness was calculated as the number of landmarks observed divided by the number of landmarks that should be observed for a specific organ. Maneuverability was calculated as the number of landmarks reached by the capsule divided by the number of landmarks that should be reached in a particular organ.

Organ	Landmarks	Completeness	Maneuverability	Overall Completeness%/Maneuverability%
**Esophagus**				93.75%/93.75%
	Upper third	15 (93.75%)	15 (93.75%)	
	Middle third	15 (93.75%)	15 (93.75%)	
	Lower third	15 (93.75%)	15 (93.75%)	
	Esophago-gastric junction	15 (93.75%)	15 (93.75%)	
**Stomach**				81.25%/87.5%
	Cardia	13(81.25%)	14(87.5%)	
	Fundus	13(81.25%)	14(87.5%)	
	Body	15 (93.75%)	15 (93.75%)	
	Antrum	15 (93.75%)	15 (93.75%)	
	Angle	15 (93.75%)	15 (93.75%)	
	Pylorus	15 (93.75%)	15 (93.75%)	
**Duodenum**				75%/75%
	Bulb	12(75%)	12(75%)	
	2nd portion	12(75%)	12(75%)	

## Data Availability

The data presented in this study are available on request from the corresponding author. The data are not publicly available due to patient privacy.
